# The Action Project Method Applied in Nursing Home Settings

**DOI:** 10.1093/geroni/igaa057.597

**Published:** 2020-12-16

**Authors:** Charlotte Jensen, Andrea Gruneir, Matthias Hoben, Jaclyn Tompalski, Adam Easterbrook, Janice Keefe, Carole Estabrooks, Sheila Marshall

**Affiliations:** 1 Staff, Edmonton, Alberta, Canada; 2 University of Alberta, Edmonton, Alberta, Canada; 3 Carlton University, Ottawa, Ontario, Canada; 4 Centre for Health Evaluation and Outcomes Sciences, Vancouver, British Columbia, Canada; 5 Mount Saint Vincent University, Halifax, Alberta, Canada; 6 University of British Columbia, Vancouver, British Columbia, Canada

## Abstract

The action-project method (APM), developed in counselling psychology and used in various disciplines, has been shown to be useful for understanding major life transitions in different contexts. We argue that the APM is beneficial for studying the impact of nursing home (NH) home admission and daily life of residents and their families/friends. The APM enables researchers to explore how residents and their families/friends experience NH-life at individual and supraindividual levels of analysis. We applied the APM to solicit the views of residents and individuals close to them to understand their priorities for quality care. The APM data collection consisted of three stages. First, a resident and family member or other caregiver met with the interviewer who initiated a conversation about their experience in the NH. The interviewer then left the room but video-recorded the conversation. Second, the interviewer met with each participant to review the video with each participant offering reflection on the original conversation. These sessions were also recorded. Following transcription and analysis of the conversations, 3 lay-language narratives were created: 1 for each individual and 1 for the pair. Third, participants reviewed their own and the pair’s narrative for additional comments. The APM offers a means to give a voice to NH residents and allows for people to talk about their experiences without the presence of a researcher. By using the APM, researchers can break down individual actions of participants and how these actions come together to form the project of navigating care in NHs.

